# 

**Published:** 1882-11

**Authors:** 


ESTABLISHED IN 1855.

OLD SERIES. Vol. XX—No. 8.

NOVEMBER, 1882.

NEW SERIES. Vol. II—No. 2.

S.G.O. INDEXED

### ATLANTA

### MEDICAL REGISTER.

(*ATLANTA MEDICAL AND SURGICAL JOURNAL.*)

EDITED BY

JAMES B. BAIRD, M.D.

H. H. DICKSON, PUBLISHER. TERMS, $2.50 A YEAR IN ADVANCE.

ATLANTA, GEORGIA:

*H. H. DICKSON, BOOK AND JOB PRINTER AND BOOKBINDER.*

1882.

